# The Composition and Physical Properties of Clots in COVID-19 Pathology

**DOI:** 10.3390/diagnostics12030580

**Published:** 2022-02-24

**Authors:** Sierk Dauwerse, Hugo ten Cate, Henri M. H. Spronk, Magdolna Nagy

**Affiliations:** 1 Department of Biochemistry, Cardiovascular Research Institute Maastricht (CARIM), Maastricht University, 6200 MD Maastricht, The Netherlands; s.dauwerse@gmail.com (S.D.); henri.spronk@maastrichtuniversity.nl (H.M.H.S.); 2 Department of Internal Medicine, Maastricht University Medical Centre+, 6229 HX Maastricht, The Netherlands; h.tencate@maastrichtuniversity.nl; 3 Thrombosis Expertise Centre (TEC), Heart+Vascular Center, Maastricht University Medical Centre+, 6229 HX Maastricht, The Netherlands; 4 Center for Thrombosis and Haemostasis, Gutenberg University Medical Center, 55131 Mainz, Germany

**Keywords:** COVID-19, hypercoagulability, ROTEM

## Abstract

Hemostasis is a finely tuned process of which dysregulation can lead either to bleeding or thrombotic complications. The latter is often caused by the hypercoagulable state as it is also seen in patients with severe acute respiratory syndrome coronavirus 2 (SARS-CoV-2) infection, i.e., in COVID-19 patients. COVID-19 patients requiring hospitalization often suffer from thrombotic events that could not be predicted using routine coagulation assays. Recently, several studies have reported ROtational ThromboElastoMetry (ROTEM) as a promising tool to predict outcomes in COVID-19 patients. In this review we give an overview of ROTEM with a particular focus on the interpretation of the symmetrical clot formation curve in relation to coagulopathy in COVID-19 patients. Furthermore, we have introduced new parameters that might help to better distinguish between COVID-19 patients and outcomes.

## 1. Hemostasis

Hemostasis is the orchestrated process of platelet reactivity and activated coagulation leading towards a stable platelet/fibrin plug formation to stop bleeding upon vascular damage. Traditionally, the process is divided into primary (e.g., platelets) and secondary hemostasis (coagulation), although various interactions between the two pathways exist.

Upon vascular damage, subendothelial collagen is exposed to blood and can capture and activate circulating platelets with the involvement of various receptors [1,2]. Stable adhesion of platelets through platelet receptor binding induces cellular signaling pathways, thereby leading to platelet activation. In general, platelet activation involves four phases: 1. a change in platelet shape to increase the surface area, 2. the release of components stored in alpha and dense granules, 3. the altered composition of the phospholipid bilayer towards a negatively charged surface rich in phosphatidylserine (PS), and 4. the activation of the GPIIb/IIIa receptor through inside-out signaling [3]. The negatively charged phospholipid bilayer facilitates the binding of the vitamin K dependent coagulation factors (discussed below), thereby enhancing coagulation and the formation of fibrin [3,4].

**Figure 1:**
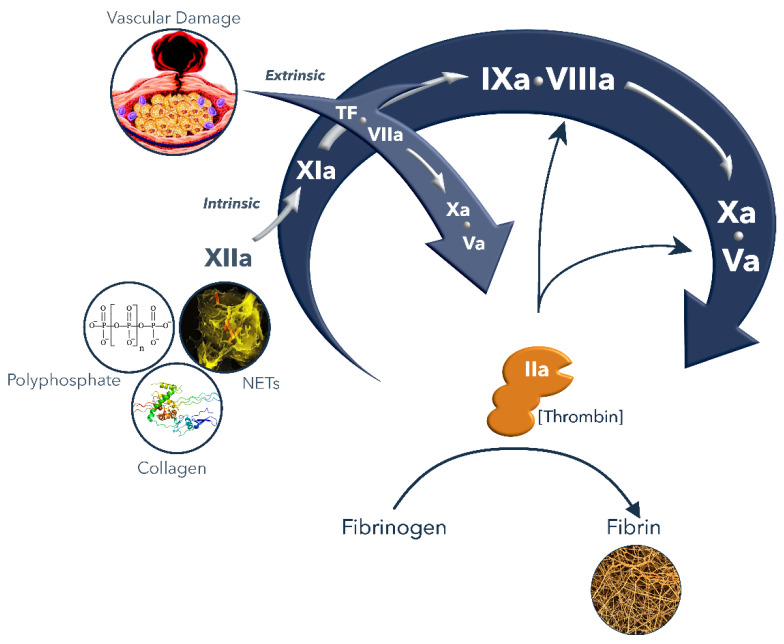
Highlight of the coagulation cascade (modified from [13]).

Secondary hemostasis encompasses the process of the formation of a fibrin clot. Upon injury of the vascular wall due to trauma, or in the case of a rupture or erosion of an atherosclerotic plaque, the subendothelial tissue factor (TF) is exposed to blood and interacts with the circulating factor VII (FVII) thereby forming a catalytic complex [5]. In a cascade mechanism, each coagulation enzyme activates a subsequent zymogen till the main enzyme thrombin (or FIIa) is formed [6] (Figure 1). Upon binding to TF, FVII undergoes auto-activation, generating FVIIa which in turn activates FX to FXa. Subsequently, FXa activates prothrombin (FII) into thrombin (FIIa). The conversion of fibrinogen into fibrin by thrombin highlights its central and major role in coagulation, along with the activation of FXIII to FXIIIa and the enhancement of its own generation through the activation of FXI and the cofactors FVIII and FV [6]. On the surface of the platelets, thrombin activates FXI to FXIa, which through the activation of FIX enhances the generation of FXa and thrombin [7]. The active cofactors FVIIIa and FVa stimulate the enzymatic activity of FIXa and FXa, thereby enhancing further thrombin generation. Thrombin converts fibrinogen into a soluble form of fibrin which then, under the influence of FXIIIa, precipitates into an insoluble fibrin mesh at the site of injury.

After the initial fibrin formation, the amplification of thrombin generation occurs as a result of positive feedback mechanisms. Therefore, more than 95% of thrombin occurs after the initial fibrin formation. Ultimately, thrombin also downregulates the coagulation cascade by the activation of the protein C pathway. Thrombin binds to thrombomodulin on endothelial cells which enhances the activation of protein C bound to the endothelial protein C receptor (EPCR), thereby generating the anticoagulant-activated protein C (APC) [8]. APC cleaves and inactivates FVIIIa and FVa, a process which requires cofactor protein S and additionally FV. Another inhibitor of the coagulation system is TFPI, which directly inhibits the main initiator of the hemostatic cascade, the TF–FVIIa complex and FXa [9,10]. The hemostatic cascade is further downregulated by serine protease inhibitors, most notably antithrombin. 

Following fibrin formation, the process of fibrinolysis regulates the fibrin network degradation to prevent unnecessary fibrin deposition. This is a serine protease-mediated process wherein the main fibrinolytic protease is plasmin that is cleaved from circulating plasminogen by either the tissue-type plasminogen activator (tPA) or by the urokinase-type plasminogen activator (uPA). Plasmin then cleaves fibrin, fibrinogen, FV, FVIII and other proteins and the proteolytic breakdown of the fibrin network can be monitored by measuring its degradation products such as D-dimer or fibrin degradation products. Plasmin generation is enhanced in the presence of fibrin and can be inhibited by natural serine protease inhibitors (i.e., serpins) such as plasminogen activator inhibitor-1 (PAI-1), α2-antiplasmin or α2-macroglobulin or by a non-serpin inhibitor, the thrombin activated fibrinolysis inhibitor (TAFI) [11,12].

## 2. ROtational ThromboElastoMetry (ROTEM) and Interpretation

Classic hemostasis assays focus on one part of the process, thereby neglecting the complex interaction between coagulation proteases, platelets, and other cells including leukocytes and red blood cells. Coagulation assays such as the prothrombin time (PT) or activated partial thromboplastin time (aPTT) are based on the time till the first fibrin fibers are formed and detected upon the activation of the system. Due to the relative high level of activation, these assays are mainly suitable for detecting deficiencies or the presence of anticoagulants (vitamin K antagonists in the PT or heparin and direct oral anticoagulants in the aPTT), but not for hypercoagulability or thrombosis risk [14,15]. The “golden” standard to assess platelet reactivity is the light transmission aggregometry (LTA) in which the light signal is blocked by platelets in plasma and transmission intensity increases following platelet aggregation upon the activation of platelets by agonists such as collagen or ADP. Again, this method is suitable to pick up platelet defects such as a deficiency in GPIb (Bernard–Soulier syndrome), GPIIb/IIIa (Glanzmann disease), or the malfunctioning of granula release, but it is not sensitive enough for evaluating platelet hyperreactivity [16,17].

**Figure 2:**
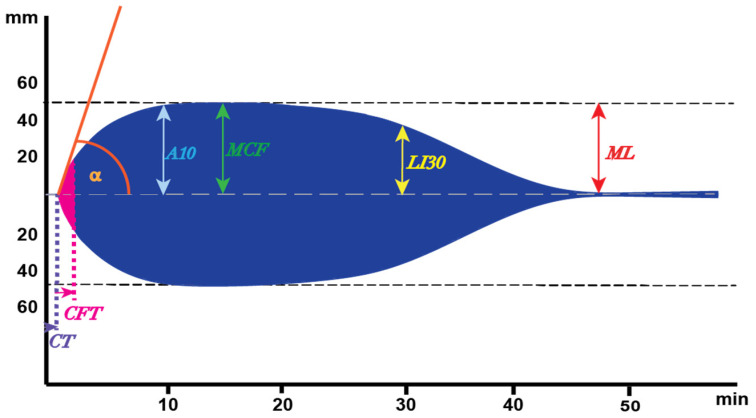
Depiction of a symmetrical ROTEM outcome. Parameters originated from the graph: Clotting time (CT): time until the 2 mm of amplitude is reached; Clot formation time (CFT): time until the amplitude of 20 mm is reached; α-angle: the angle between the baseline and the tangent to the curve via the 2 mm amplitude point; A10: amplitude at 10 min; Maximum clot firmness (MCF): maximum amplitude; LI30: clot lysis index at 30 min; ML: maximum lysis.

To overcome the limitations of focused assays, laboratory methods have been developed to incorporate the almost complete coagulation cascade such as in plasma thrombin generation methods or overall methods that include all blood cells and measure the elasticity of the thrombus formed. Of the latter type, the two main methods are ThromboElastoGraphy (TEG) and ROtational ThromboElastoMetry (ROTEM) in which a pin is suspended in a cup with whole blood. Rotation is involved in both methods with the main difference that in TEG the cup rotates and in ROTEM the cup is fixed with a rotating pin [18,19]. Irrespective of whether the cup or pin rotates, some impediment to the rotation develops as the blood forms a platelet-rich fibrin thrombus. The degree of this impediment is recorded as amplitude (A) and expressed in mm against time in the symmetrical amplitude graph [20]. The pin is connected to an axis that symmetrically oscillates to its left and right. The axis is supported by a high precision ball bearing and is driven by a motor which is connected via an elastic spring. The impedance of rotation is detected through a diode light source attached to the axis and mirrored for reflection towards a charged coupled device (CCD) sensor. Clotting, i.e., fibrin formation, in the sample obstructs movement of the pin, which is then observed via deviations in optical perception, which are processed and displayed graphically [18,19]. The outcome, a symmetric “cigar” looking graph (Figure 2) is generated by plotting the time on the horizontal axis and the amplitude of the clot on the vertical axis. The magnitude of this mirrored symmetrical reaction curve is proportional to the strength of the clot formed over time. This method of data representation allows the characterization of the clot formation.

In a ROTEM assay, the coagulation activity can be triggered by various factors, e.g., through the addition of tissue factor for the extrinsic route (EXTEM) or by an activator of factor XII for the intrinsic pathway (INTEM). Alternatively, the method can be modified to be dependent on the endogenous activators (NATEM) or independent of the platelets (FIBTEM) [18,21].

A typical ROTEM graph is depicted in Figure 2 and the parameters derived from the graph are shown. Clotting Time (CT), the initiation of the clot formation, indicates the time until the first traces of fibrin are formed, and the clotting is marked with a 2 mm amplitude. In principle, the CT is comparable to the traditional PT or aPTT, where the time represents the first traces of fibrin generated as well. The next part of the graph is from the 2 mm amplitude to the 20 mm mark representing the Clot Formation Time (CFT), which is the time needed for fibrin formation to increase from a 2 mm to 20 mm amplitude. At the 2 mm amplitude point, angle α represents the angle between the tangent to the curve and the baseline. This angle is obtained by drawing a tangential line from the point (2 mm) where the symmetrical curve reaches the 20 mm amplitude to both sites. The higher the angle, the higher the amount of fibrinogen present in a sample and the prolongation of this angle may indicate a shortage of platelets, platelet dysfunction, fibrinogen deficiency, or malfunction. The maximum of the curve is known as the Maximum Clot Firmness (MCF) in mm and the time until this moment is called the Maximum Clot Firmness time (MCF-t). Upon reaching the maximum clot formation, and most likely started with the first amounts of thrombin generated, fibrinolysis becomes visible by the decline in clot firmness until the curve is back to the baseline and all fibrin is degraded into fibrin degradation products. Besides the Maximum Lysis (ML) upon reaching the baseline and describing the difference between MCF and the lowest amplitude, fibrinolysis is characterized by another main parameter, the Lysis Index (LI), 30 min after the CT in percentages. This is calculated by dividing the amplitude at that moment by the MCF times 100% [18,19,22,23].

## 3. Coagulopathy in COVID-19

Coronavirus disease 2019 (COVID-19) is a severe acute respiratory syndrome coronavirus 2 (SARS-CoV-2) infection associated with severe inflammatory and thrombotic conditions. The prediction of COVID-19-associated thrombotic events, however, is challenging as routine coagulation assays cannot recognize COVID-19 patients at risk for thrombotic complications [24,25]. For instance, only small changes in prothrombin time (PT) and no changes in activated partial thromboplastin time (aPTT) have been reported in COVID-19 patients [25,26]. Yet, COVID-19 is highly associated with thromboembolic complications and up to 25% of patients at the intensive care unit suffer from such events [24,27]. The majority of these thromboembolic complications consist of venous thromboembolism, with a high prevalence of pulmonary embolisms, but arterial events (myocardial infarction, ischemic stroke) have also been associated with COVID-19 infections [28,29,30]. While the coagulation abnormalities in COVID-19 patients were thought to manifest into disseminated intravascular coagulation (DIC) as often seen in other severe systemic inflammatory diseases, COVID-19-induced coagulopathy displayed distinct biochemical properties and clinical phenotypes [31,32,33,34].

Strikingly, thromboembolism occurs in spite of the administration of anticoagulant medication (mainly (low molecular weight) heparins), which raised questions regarding the optimal management of these patients [35]. Notably, baseline cardiovascular complications associated with hypercoagulability (e.g., atrial fibrillation or kidney dysfunction) are more prevalent in COVID-19 patients with thrombotic complications which may further contribute to the worsening of hypercoagulability in COVID-19 patients [36]. The overall effect of combined pathologies on hypercoagulability and outcomes in global assays such as the ROTEM needs to be elucidated in further clinical studies. In addition to this hypercoagulability, COVID-19 coagulopathy is also characterized by excessively high levels of fibrinogen and markers of endothelial activation, including the von Willebrand factor, platelet activation, and increased proteolysis of fibrin (evident as d-dimer fragments), while fibrinolysis overall appears to be insufficient to prevent widespread fibrin deposition (relative insufficiency of fibrinolysis) [37,38,39,40]. These abnormalities might also influence the global coagulations assays used to assess coagulopathy. For these reasons, the viscoelastic methods, i.e., TEG, ROTEM analysis, appear to be an interesting technology to study both fibrin formation and lysis in these patients, with the hope of being able to use test outcomes to optimize antithrombotic therapy [41,42]. Specifically, the ROTEM variables MCF (maximum clot firmness) CT (clotting time), and CFT (clot formation time) have been studied as predictors of thromboembolic complications [43,44,45,46,47].

Elevated levels of D-dimer have been reported as strong prognostic factors for poor outcomes in COVID-19 patients [25,48]. However, the application of D-dimer levels as prognostic biomarkers in the early stages of disease is not commonly used throughout infection [48]. Therefore, alternative assays are explored and the maximum clot firmness (MCF) variable of ROTEM appears to be a promising marker for hypercoagulability in COVID-19 [49].

**Figure 3:**
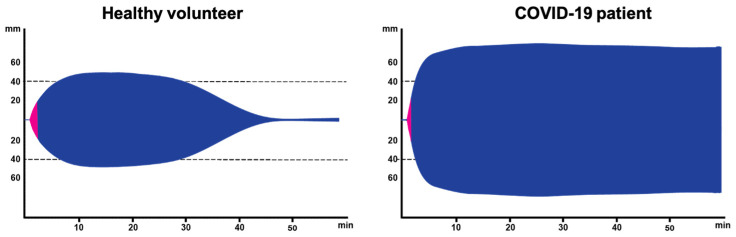
Representative example of a ROTEM outcome in a healthy volunteer and in a COVID-19 patient.

The ROTEM outcome is markedly different in a COVID-19 patient when it is compared with the results of a healthy volunteer (Figure 3), as indicated by an explosive generation of fibrin with a strongly elevated MCF resulting in a wider symmetrical ROTEM outcome. This could probably be explained by the very high fibrinogen levels in the blood of COVID-19 patients. In addition, no lysis is detectable in the COVID-19 samples as the clot remains present during the whole time-course of measurement. In line with these differences, van den Berg and Hulshof investigated the effect of the higher fibrinogen levels in severely ill COVID-19 patients to address questions related to the clinical observation of resistance to anticoagulant treatment in COVID-19 patients while at the ICU department [50]. In this study, blood from healthy volunteers was spiked with anticoagulants (i.e., heparins) and/or fibrinogen to mimic the characteristics of the COVID-19 samples (collected from the ICU department) with the aim of dissecting the relative contribution of these factors to the ROTEM outcome. It appeared that heparin addition increased the CT that could return to normal level in the presence of a high fibrinogen level. In contrast, heparin did not affect MCF, whereas the addition of fibrinogen resulted in an increased MCF that was not affected by heparin addition. Given that the combination of high fibrinogen and heparin may resemble the situation in the COVID-19 patients and similar changes are expected, ROTEM appeared to be a valuable approach for measuring COVID-19 samples [50].

## 4. Novel Parameters Originated from ROTEM

**Figure 4:**
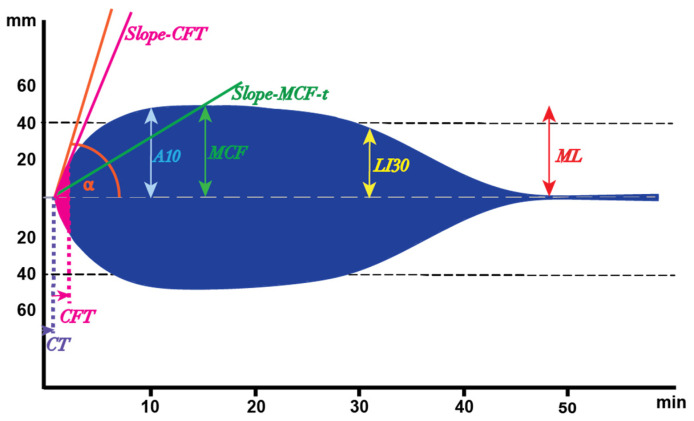
Novel parameters, slope-CFT and slope-MCF-t for better characterizing the ROTEM outcome. Slope-CFT is the slope between the start of the CFT and the end of the CFT and Slope-MCF-t represents the slope between the 2 mm amplitude and the MCF. The conventional parameters (e.g., CT, CFT, etc.) have been explained in Figure 2.

Despite the entirely different appearance of the ROTEM results as seen in COVID-19 patients (Figure 3), the ROTEM parameters cannot describe all aspects of the differences. For instance, the rate of the clot formation is not described in the traditional analysis, whereas it is apparent in Figure 3 that the maximum clot formation occurs at a higher rate. Therefore, we re-analyzed the ROTEM data reported in the previous study [50] to introduce two novel parameters that might give a better insight into the clot formation in COVID-19 patients. The first parameter was defined as the slope between the start of the CFT until the end of the CFT and was defined as the Clot Firmness Increase (CFI) from the 2 mm amplitude to the 20 mm amplitude (Slope-CFT, Figure 4). The second parameter, Slope-MCF-t (in mm/min, Figure 4), was defined as the increase in CFI from the 2 mm amplitude to the MCF (in mm/min, Figure 4).

**Figure 5:**
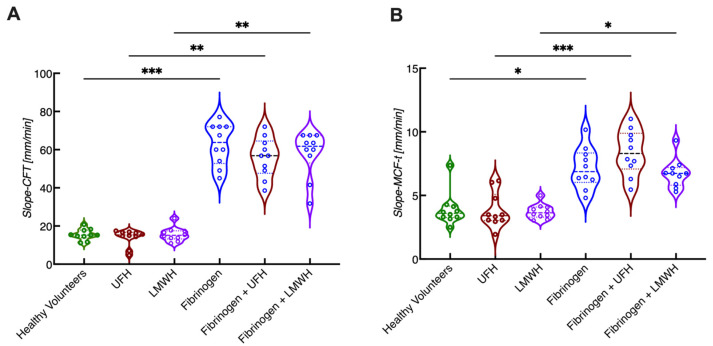
Comparison of new ROTEM parameters slope-CFT and slope-MCF-t derived from in vitro spiking experiments. Whole blood samples were obtained from healthy volunteers and spiked with anticoagulants (unfractionated heparin, UFH or low molecular weight heparin, LMWH) and/or fibrinogen. (See experimental details in [50]). Slope-CFT (**A**) and slope-MCF-t (**B**) are shown. *n* = 10, * *p* < 0.05, ** *p* < 0.01, *** *p* < 0.001.

Comparing the slope-CFT and slope-MCF-t in whole blood samples with or without additional spiking of fibrinogen and/or heparins, it appeared that both the slope-CFT and slope-MCF-t were significantly higher upon fibrinogen spiking (Figure 5). These increased values did not change upon the addition of heparin, showing that these processes are indeed mainly determined by the high levels of fibrinogen in an in vitro setup.

**Figure 6:**
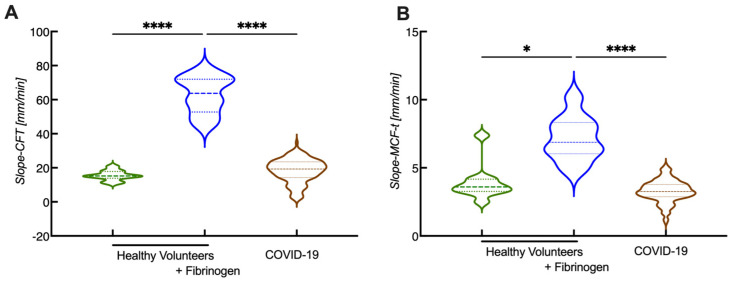
Comparison of slope-CFT and slope-MCF-t in samples from in vitro spiking experiments and in samples from COVID-19 patients. Whole blood samples were obtained from healthy volunteers and spiked with fibrinogen and from COVID-19 patients. (See experimental details in [45,50]) Slope-CFT (**A**) and slope-MCF-t (**B**) are shown. N_Healthy volunteers_ = 10, N_COVID-19_ = 141, * *p* < 0.05, **** *p* < 0.0001.

After characterizing the data from the in vitro spiking experiments, the new calculations for CFI were also applied on COVID-19 data to see whether the COVID-19 patient results were comparable to the fibrinogen-spiked samples of healthy volunteers. Applying the new parameters on COVID-19 samples revealed that slope-CFT was only slightly increased compared with the healthy volunteers (Figure 6A), while the slope-MCF-T showed no difference (Figure 6B). In contrast, samples from healthy volunteers spiked with fibrinogen resulted in an over 3-fold increase in slope-CFT and slope MCF-t (Figure 6). It seems that the rate of clot formation is highly dependent on the fibrinogen content. Given that the COVID-19 patients have high fibrinogen levels, one could expect a similar outcome; however, this was not the case. Hence, the presence of other rate-limiting factors has a bigger impact on the rate of clot formation.

## 5. ROTEM beyond COVID-19

While hypercoagulability has received much attention recently due to its profound role in COVID-19, it has also been long associated with several other cardiovascular pathologies (e.g., DVT, atrial fibrillation, stroke, etc.) but it has also been associated with non-cardiovascular pathologies such as malignancies [51]. ROTEM analysis has shown the ability to identify hypercoagulable states in patients undergoing major surgery or with malignancies [49,52]. Moreover, patients with an increased risk for thrombotic complications, i.e., with hypercoagulability are often receiving thromboprophylaxis that interferes with the coagulation assays and requires monitoring. Importantly ROTEM has shown a good sensitivity for monitoring thromboprophylaxis in patients receiving LMWH and DOACs and also in vitro [53,54,55,56].These findings are suggestive for the promising role of ROTEM as a novel promising monitoring tool; however, further DOACs specific validation will be required for implementing ROTEM in daily clinical practice [57].

## 6. Discussion and Future Perspectives

COVID-19 is highly associated with thrombotic complications that are unpredictable in terms of occurrence and severity. This poses a major clinical problem as regular thromboprophylaxis fails to sufficiently protect COVID-19 patients with a persistent rate of venous thromboembolism by up to 25% in the most severely affected patients. While conventional, coagulation assays, such as prothrombin time (PT) or activated partial thromboplastin time (aPTT) do not correlate with the COVID-19 outcome, ROTEM has been shown to be a promising approach for this purpose [26,41]. 

The advantage of ROTEM is that it incorporates whole blood and depending on the trigger, different aspects of coagulation activity can be studied. The classical parameters of ROTEM (e.g., CT, MCF, MCF-t) have been shown to be associated with the outcome of COVID-19 to some extent; however, it does not reflect well on the increase in clot firmness (CFI) [31,32,33,34]. CFI could maybe better explain the underlying mechanism behind the changes in COVID-19 patients. Therefore, we have introduced two new parameters, the slope-CFT and slope-MCF-t to characterize the CFI that may provide a better explanation regarding the rate of the clot formation. 

Interestingly, the maximum clot firmness in COVID-19 patients was comparable to that of the fibrinogen-spiked samples of healthy volunteers, while the growth of the clot firmness (CFI) was not comparable to that of the fibrinogen-spiked samples of healthy volunteers. Therefore, other components must account for this phenomenon, or the combination of several components could influence the CFI. There are some possible explanations: (1) maybe the heparin had some effect in the COVID-19 patients, stalling the fibrin formation; (2) fibrinolysis might also have been triggered in COVID-19 patients, more than in the spiked samples, breaking down the fibrin constantly, while simultaneously there was enough fibrinogen and thrombin present to build up new fibrinogen as well; (3) the high concentration of D-dimers in the COVID-19 samples triggered coagulation starting the clot formation simultaneously with the lysis of the clots. However, these speculations require testing in further research.

More in-depth knowledge about the clot formation in COVID-19 samples could provide solutions in treatment of hypercoagulability and hyperfibrinolysis, for example, in COVID-19 patients. New parameters describing CFI and slope-CFT might give more meaning to the CFT, and the slope-MCF-t could provide a better understanding of the MCF as it describes the rate of the clot formation. Further validation of these novel parameters is required to fully elucidate their importance.

## Data Availability

Original data are available upon requests.
